# Hybrid spin-crossover nanostructures

**DOI:** 10.3762/bjnano.5.232

**Published:** 2014-11-25

**Authors:** Carlos M Quintero, Gautier Félix, Iurii Suleimanov, José Sánchez Costa, Gábor Molnár, Lionel Salmon, William Nicolazzi, Azzedine Bousseksou

**Affiliations:** 1 LAAS, CNRS & Université de Toulouse (UPS, INSA, LAES), 7 Av de Colonel Roche, 31077 Toulouse, France; 2 LCC, CNRS & Université de Toulouse (UPS, INPT), 205 route de Narbonne, 31077 Toulouse, France; 3 Department of Chemistry, National Taras Shevchenko University of Kiev, 62 Volodymyrska St. 01601, Ukraine

**Keywords:** core–shell particle, multifunctionality, nanomaterials, spin-crossover

## Abstract

This review reports on the recent progress in the synthesis, modelling and application of hybrid spin-crossover materials, including core–shell nanoparticles and multilayer thin films or nanopatterns. These systems combine, often in synergy, different physical properties (optical, magnetic, mechanical and electrical) of their constituents with the switching properties of spin-crossover complexes, providing access to materials with unprecedented capabilities.

## Review

### Introduction

More than 15 years ago, Olivier Kahn highlighted the great potential of the so-called spin-crossover (SCO) materials on the nanoscale [1]. Indeed, there are interesting fundamental questions with regards to the size effect on the phase transition temperature, on the hysteresis width, on the kinetics of the spin transition, etc. On the other hand, SCO nanomaterials are also attractive candidates for integration into a variety of emerging micro- and nano-technologies. The notable characteristics of SCO materials include: i) reversible changes in their various physical properties (magnetic, optical, electrical and mechanical), ii) diverse external stimuli to drive their transition, iii) their versatility, i.e., there are multiple complexes with different transition properties, iv) room temperature operation and v) their bistability can be kept down to the nanoscale.

In the last few years, diverse pathways for the production of SCO nanomaterials as colloidal suspensions, thin films and other types of nanoscale assemblies have been established using different chemical and/or lithographic approaches for controlling the size, shape and even the organization of SCO nano-objects [2–11]. Furthermore, there is an active quest for developing novel methods which are sensitive enough to probe extremely small quantities of SCO materials for a better understanding of these materials at the nanoscale. All of these recent results have been extensively reviewed in [12–16]. In the present review, we focus on new types of emerging, hybrid nano-objects that involve SCO nanomaterials in complex structures, which reveal unique functionalities due to the synergy between the SCO properties and the physical properties (magnetic, photonic, charge transport, etc.) of the surrounding matter. The present review constitutes an overview of these systems including their synthesis, theoretical modelling and future possible technological applications.

Indeed, a recent strategy to access the multifunctional potential of novel nanomaterials was the development of nanohybrid or nanocomposite structures that are able to combine different materials with different properties. Typically, in this approach, at least one of the components is organic while the other is inorganic in nature. A nanocomposite is a multiphase solid material where one of the phases has one, two or three dimensions in the size range of 1–100 nm. Additionally, it is worth noting that the molecular building blocks that constitute these hybrid materials can be as big as inorganic clusters, typically in the nanometer range. The most notable advantage of controlling their mutual arrangement is that they can effectively combine the properties of both components into one material with the additional possibility to present synergetic effects, and thus properties which were unattainable in the constituent parent materials [17]. The properties of these hybrid structures are not only interesting from a fundamental point of view, but are currently envisaged to be applied in various fields of technology.

### Synthesis of hybrid SCO nanostructures

**Figure 1:**
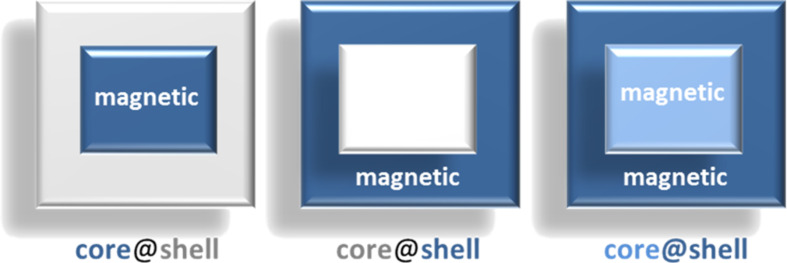
Classification of core–shell SCO systems.

The development of functionalized nano-composite materials with potential applications in the field of switchable materials has recently attracted great attention mainly due to the development of hybrid nanoparticle molecules (HNMs) [18] and hybrid nanoparticle-coordination network structures (HNCNSs) [19]. Here, some remarkable examples of sophisticated structures involving SCO activity recently appeared in the literature and are examined according to a simple classification based on the position of the active SCO species on the core–shell nanostructure. One can thus envision a case in which the switchable active species is placed at the core, a second type where the active species is at the shell, and finally, a third type where both the core and the shell substructures are active (see Figure 1).

**Figure 2:**
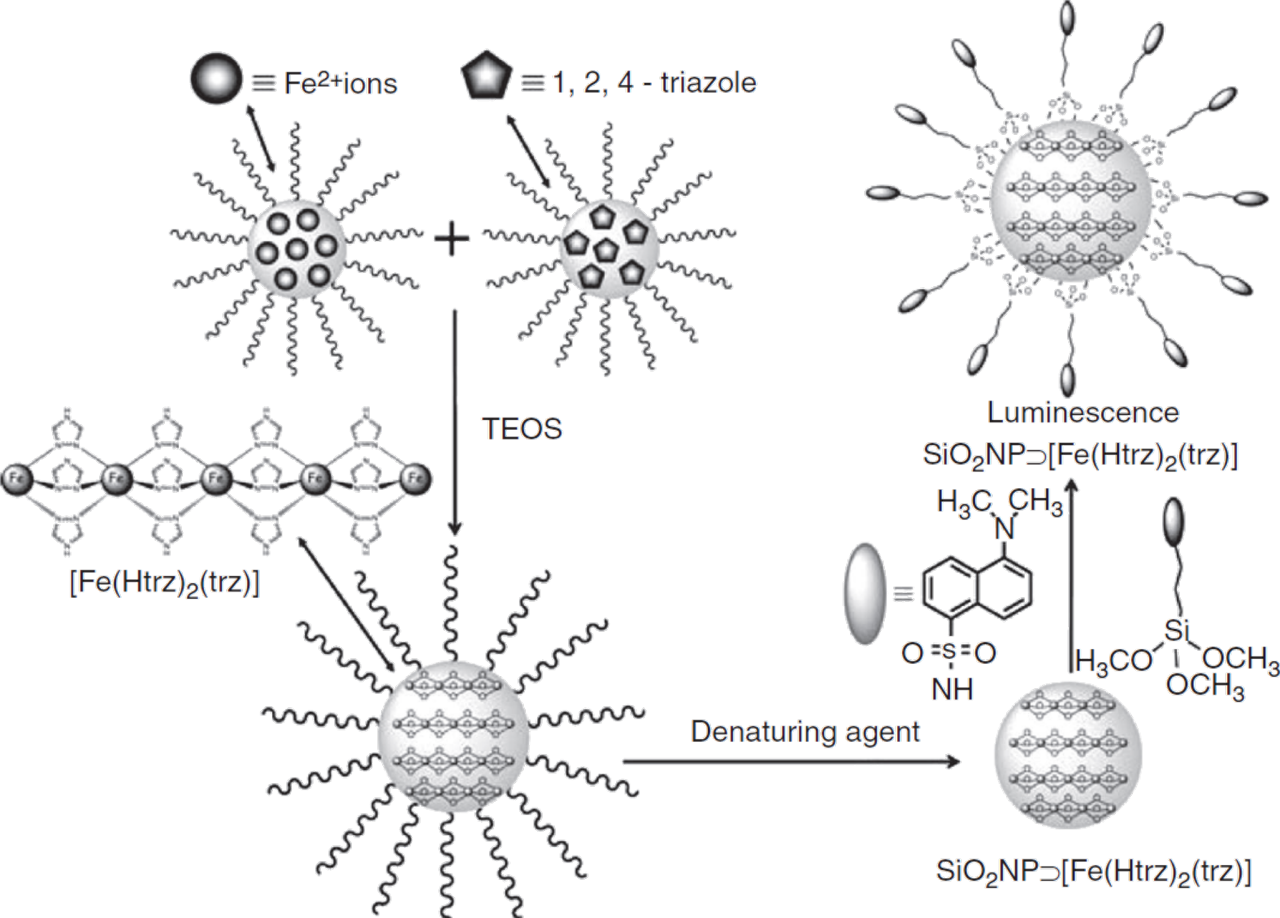
Synthesis route and schematic representation of the luminescent, SCO, SiO_2_ nanoparticles. Reproduced with permission from [21], copyright 2011 Wiley-VCH.

To our knowledge, for the first type, only three examples have been reported. Raza et al. [20] produced core–shell nanostructures based on a Hofmann-type clathrate SCO core with different shell materials. Although they observed a very interesting effect of the shell thickness on the SCO properties, their study did not aim for development of multifunctional materials. Titos-Padilla et al. [21] reported a core–shell nanocomposite with a SCO core synthetized from the coordination polymer [Fe(Htrz)_2_(trz)](BF_4_) (Htrz = 1,2,4-triazole and trz = 1,2,4-triazolato), known to show a memory effect above room temperature [22], and SiO_2_ shell grown around it. The use of silica is of great interest because of its high porosity and the possibility of grafting other functionalities onto its surface. In this case, the luminophore 3-(dansylamido)propyltrimethoxysilane was grafted onto the surface of the nanoparticles (NPs) using a straightforward chemical reaction (see Figure 2). The luminescent signal from these core–shell particles during the thermal cycles follows the SCO curve obtained from magnetic studies. Consequently, the authors affirm that the grafting process did not significantly affect either the morphology or the magnetic properties of the NPs. In this system, the luminescent signal from the dansyl is quenched by the Fe(II) low-spin state (LS) centers of the coordination polymer as a consequence of the spectral overlap between the dansyl emission and the absorption band of the LS ions.

Following this synthetic strategy, a new nano-composite material with the same SCO complex {[Fe(Htrz)_2_(trz)](BF_4_)} was reported by our team, which associated the SCO complex with gold NPs by means of an intermediate decorated silica shell [23]. Briefly, SCO@SiO_2_ particles were synthetized using the reverse micelle technique by mixing two microemulsions: one containing the triazole ligand and the other the iron(II) salt, using Triton and TEOS (tetraethoxysilane) as tensioactive and silica sources, respectively. The SCO nano-composite particles were combined with gold NPs with the aim of using the properties of the gold to absorb light and convert it to heat (via a strong photothermal effect). Despite the small volume fraction of gold NPs within the nanocomposite (around 0.5%), the laser power required for a complete spin-state switching process was reduced by around 70%. It is interesting to note here that a similar strategy for low power, laser switching was also developed using the strong infrared absorption of the polymer matrix in a polymer–SCO composite material [24]. This work revealed exciting applications for high density read/write optical memory devices based on SCO compounds.

**Figure 3:**
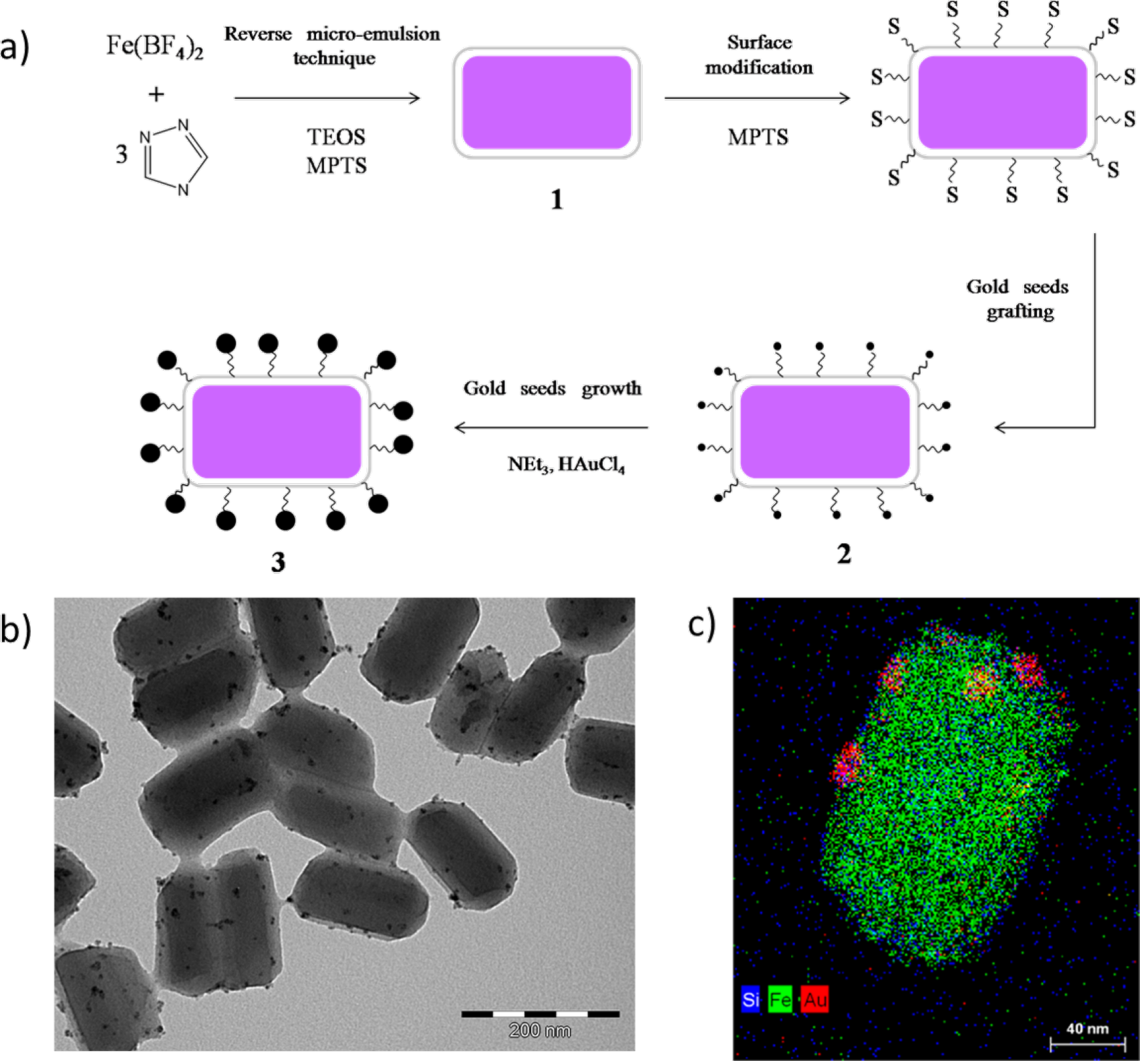
a) Schematic overview of the formation of the nanocomposite, gold-decorated SCO–SiO_2_ nanoparticles. b) TEM and c) STEM–EDX images of the particles. Adapted with permission from [23], copyright 2014 The Royal Society of Chemistry.

As far as we know, the second and third types of core–shell structures were only achieved thus far by using Prussian blue analog complexes (PBA). While not all of these compounds are switchable, some can exhibit a charge transfer-induced, SCO phenomenon.

**Figure 4:**
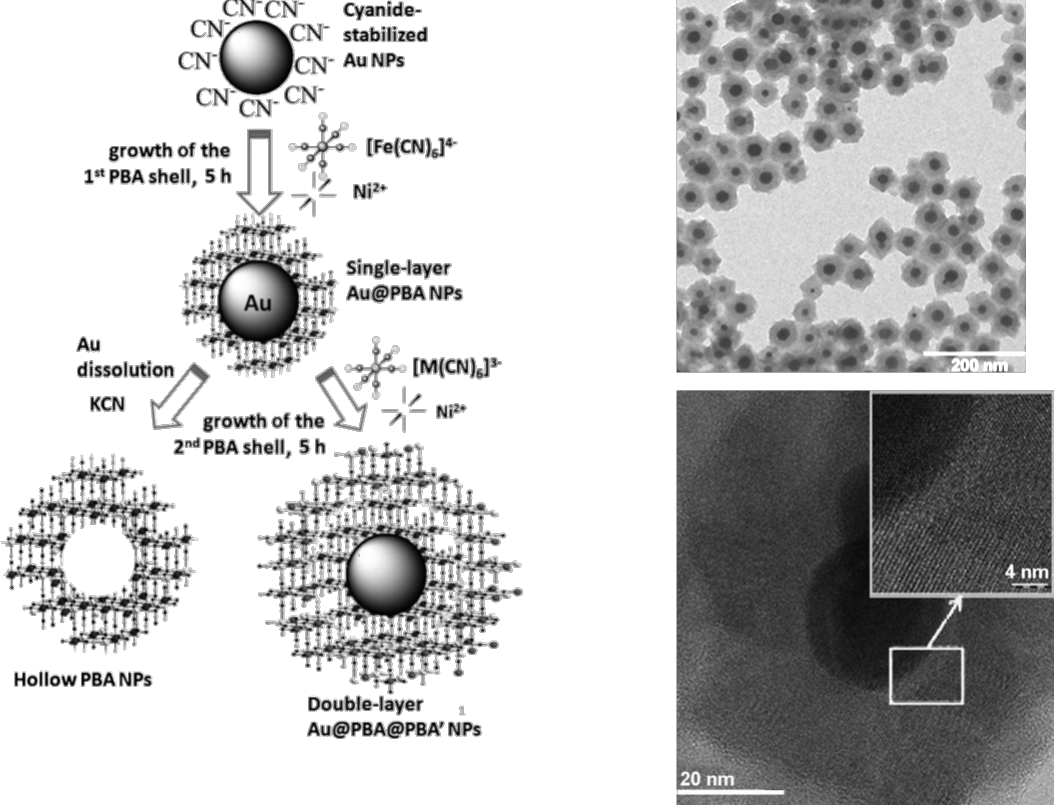
Schematic representation of single layer Au@PBA nanoparticles, double layer Au@PBA@PBA core–shell NPs, and hollow PBA NPs. On the right, TEM and HRTEM images of the Au@KNiFe NPs. Adapted with permission from [25], copyright 2014 Wiley-VCH.

Guari et al. [25] has described a practical approach for the synthesis of single layer Au@PBA and double layer Au@PBA@PBA core–shell NPs. The synthesis route developed by the authors is a two-step process: first, an aqueous synthesis of cyanide gold NPs was performed and second, a cyano-bridged polymer shell was grown on the surface of the Au NPs by controlling the time of the reaction process. Following these procedures, two more remarkable processes were achieved: a second layer of PBA was inserted into the previous architecture in a controlled manner and additionally, the gold was removed from the core (Figure 4). As expected by the authors, the new NPs containing a gold core display both properties: the plasmonic optical property provided by the gold and the magnetic interactions from the PBA compound on the shell. In summary, the single layer NPs seem to behave differently from the double layer nanostructures. Namely, the single layer NPs exhibit a paramagnetic behavior while the double layer NPs exhibit ferromagnetism. Therefore, these new hybrid materials may be considered as multifunctional.

**Figure 5:**
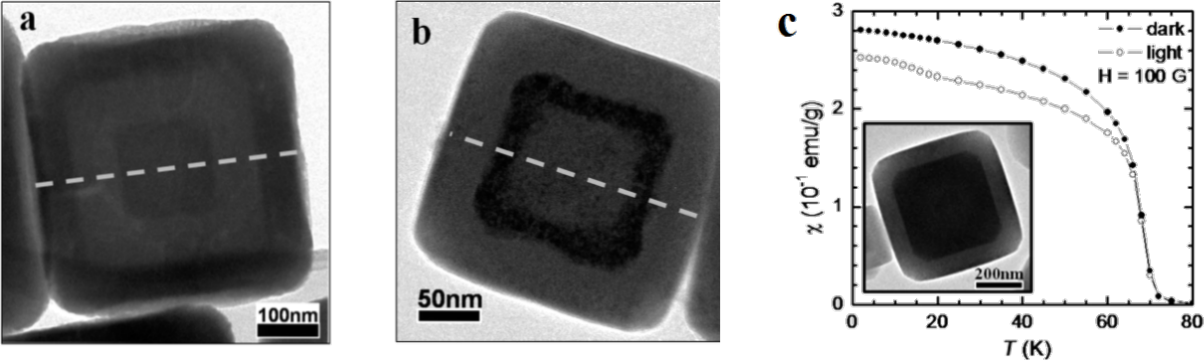
HRTEM images of core–multishell PBA nanoparticles a) RbCoFe@KNiCr@RbCoFe and b) KNiCr@RbCoFe@KNiCr, and c) shows the field-cooled magnetic susceptibility as a function of temperature before and after light irradiation of RbCoFe@KNiCr. Adapted with permission from [27], copyright 2011 American Chemical Society.

The third type of core–shell or core–multishell NPs contains two active magnetic species. Catala and Mallah reported that it is possible to carry out epitaxial growth of a 3D PBA different from that used for the core in the case of 10 nm Cs[Ni^II^Cr^III^(CN)_6_]@Cs[Co^II^Cr^III^(CN)_6_] heterostructures [19] or 50 nm Cs^I^[Co^II^Cr^III^(CN)_6_]@Cs^I^[Fe^II^Cr^III^(CN)_6_]@Cs^I^[Ni^II^Cr^III^(CN)_6_] systems [26]. These core–multishell coordination nanoparticles were fabricated using a straightforward, surfactant-free manipulation with precise size control of the sample by controlling the addition rate and the concentration of the components. It is worth noting that these new combined materials present a different magnetic behavior than the associated pure nanoparticles. The authors attributed this result to a synergetic effect between the different ultrathin shells, allowing a modulation of the magnetic response of the nanoparticles. This method was also used by Talham to study the photoinduced switching of the magnetism of K_j_Ni_k_[Cr(CN)_6_]_l_@Rb_a_Co_b_[Fe(CN)_6_] heterostructures [27]. These nanoscale heterostructures exhibited a photo-response not seen in either constituent on its own (Figure 5). The changes induced by the light irradiation occur in the RbCoFe lattice, which experiences a charge transfer-induced spin transition from the Fe^II^-CN-Co^III^(LS) to the Fe^III^-CN-Co^II^(HS) state. According to the authors, the increase in the volume during this photo-switching process modifies the magnetism of the inner KNiCr layer due to magnetostrictive effects.

### Devices based on hybrid SCO nanostructures

#### Luminescent devices

**Figure 6:**
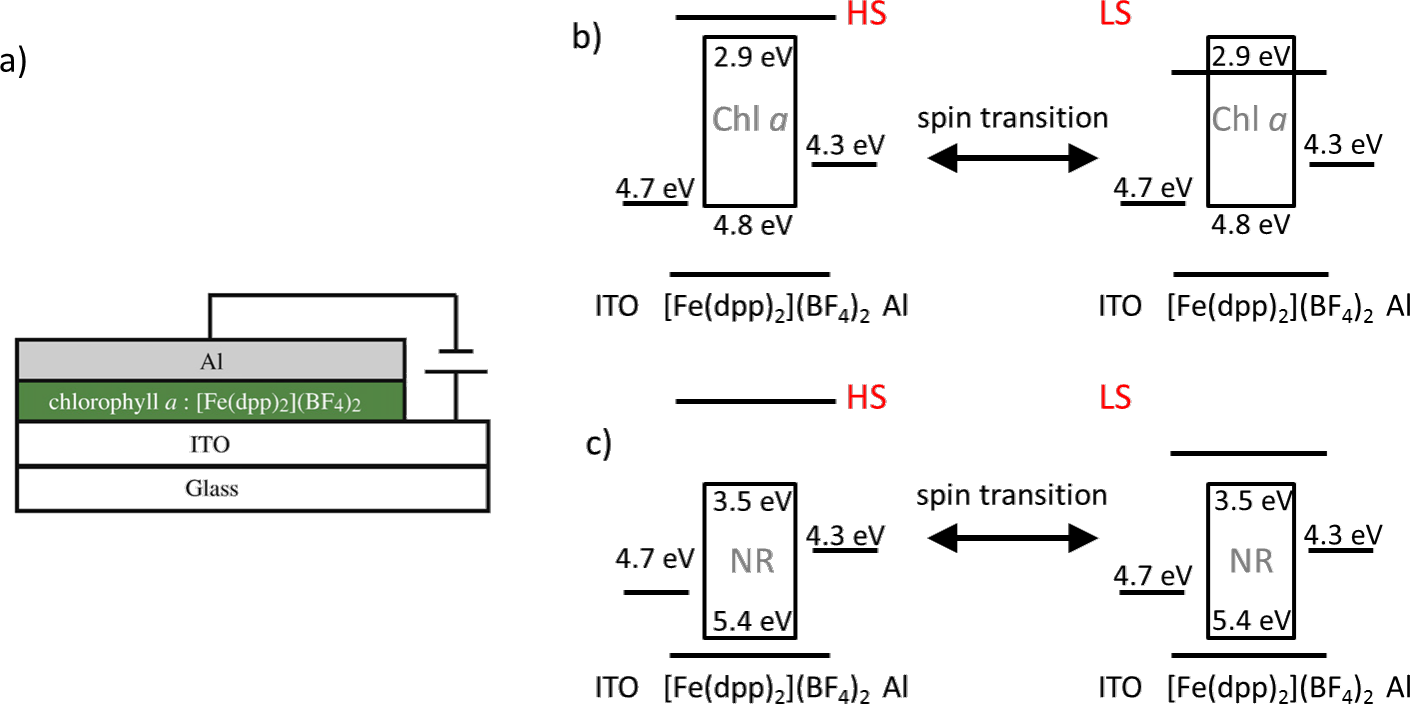
a) Architecture of the OLED device constructed by Matsuda et al. [28]. b) A schematic representation of the mechanism proposed for the EL, on/off switching, based on an energy level diagram of a device using Chl *a* and [Fe(dpp)_2_](BF)_4_. c) Energy level diagram for an analog device but employing NR as the emitting material [30]. Adapted with permission from [28] and [30], copyright 2008 and 2013 Elsevier.

Matsuda et al. proposed a synthesis strategy that exploits the synergy between the charge carrier orbitals of a SCO complex and a light emitting material. They developed a concept for an organic light emitting diode (OLED) that consists of a 50 nm light emitting thin film composed of chlorophyll *a* (Chl *a*) mixed with the SCO complex [Fe(dpp)_2_](BF)_4_ (dpp = 2,6-di(pyrazol-1-yl)pyridine) spin-coated on an indium tin oxide (ITO) substrate (anode) and then covered by a 30 nm thick Al cathode (see Figure 6a) [28]. With this configuration, the electroluminescence (EL) of the device can be reproducibly switched on/off as a function of temperature due to the thermal spin state switching. Indeed, the light emission of this type of OLED is severely quenched if the [Fe(dpp)_2_](BF)_4_ is in its LS form (*T* < 260 K). After a photoluminescence study of identical films of [Fe(dpp)_2_](BF)_4_ and Chl *a,* the authors excluded the possibility of an energy transfer from the excited Chl *a* to the SCO complex in the LS state. These observations suggests that the excited form of Chl *a* does not exist in the OLEDs at low temperatures [29].

To explain these findings, the authors proposed two mechanisms: first, since the oscillator strength of the charge transfer (CT) bands increases in the LS state, it is possible that the injected electrons transit from the π orbital of the dpp ligand to the d orbital of the iron centers, giving an additional electron transport path from the cathode to the anode through the SCO complex. Second, a shift in the energy level of the molecular orbital concerning the electron transport in the SCO complex relative to that of Chl *a* (Figure 6b) [30] is possible. Thus, at high temperatures (HS state) the injected electrons effectively excite the Chl *a* molecules, leading to EL emission. Conversely, at low temperatures (LS state) the electron transport orbital of the [Fe(dpp)_2_](BF)_4_ shifts to a level lower than that of Chl *a* and as a result, the electrons flow exclusively into the SCO complex, preventing the formation of excited Chl *a*. Even though the shift of this orbital in the SCO complex is unknown, the authors confirmed their model by changing the light emitting compound to Nile Red (NR). This dye presents an electron transport orbital below that of Chl *a*; in consequence, in spite the change of the electronic configuration of the [Fe(dpp)_2_](BF)_4_, the EL emission persists at all temperatures (Figure 6c).

In a different approach, the luminescent response of a luminophore can also be effectively quenched if its emission/excitation displays an adequate spectral overlap with one of the distinctive LS or HS absorption bands of a SCO complex. In this manner, the luminescence will be modulated as the complex switches its spin state. Nonetheless, in order to render this approach valuable at the nanoscale, it is imperative to place the luminophore close to the metallic centers of the complex (≈1–3 nm) to establish a non-radiative energy transfer [31]. In our research, we exploited this idea by using an acridine orange dye as the luminescent doping agent for the SCO complex Fe(hptrz)_3_(OTs)_2_ (hptrz = 4-heptyl-1,2,4-triazole) [32]. In addition to its strong emission in the green spectral range that overlaps with the characteristic LS absorption band of the SCO complex (centered at 543 nm), these molecules may serve as ligands in substitution for hptrz and thus, they are likely to approach the Fe(II) centers during the synthesis. Regular arrays of luminescent, SCO, nano-objects with an average size of 200 × 150 nm were patterned by employing a nano-patterned polydimethylsiloxane (PDMS) stamp. The luminescence of the isolated dots as a function of temperature increased upon the LS to HS spin transition and decreased as the LS state was restored at low temperatures. The synergy between luminescence and SCO properties in these hybrid systems also has interesting potential for thermal imaging applications. Molnár et al. demonstrated that thin films of the system as employed in [32] can be successfully used as luminescent surface temperature sensors with high spatial resolution [33]. The greatest benefit from this luminescent, SCO-based probe lies in the fact that the speed and temperature range (where the spin transition takes place) can be tuned by well-known chemical synthesis methods and without necessarily modifying the luminescent agent. This can be translated into a flexible design when it comes to sensitivity and working range of the probe at fixed wavelengths.

#### Active plasmonic devices

**Figure 7:**
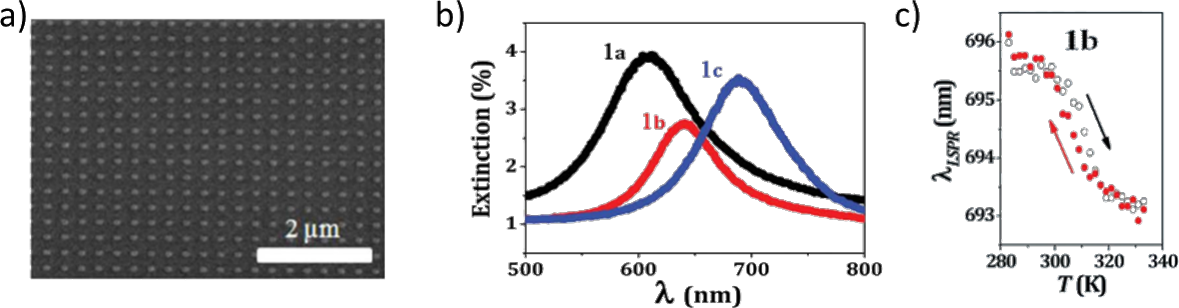
a) SEM image of a gold nanorod array with 200 nm pitch. b) Extinction spectra of three nanorod arrays with different aspect ratios. c) Plasmon resonance shift associated with the spin crossover of a 60 nm thin film deposited onto the nano-dot array displayed in a) as a function of temperature. Adapted with permission from [34], copyright 2013 The Royal Society of Chemistry.

Currently, one of the most dynamic research area in the nanosciences is plasmonics. Surface plasmons provide unprecedented capabilities for manipulating electromagnetic waves at the nanoscale and have opened the door to unique photonic applications involving biological/chemical sensors, signal processing and solar energy harvesting. In particular, emerging, active, plasmonic devices employ hybrid nanostructures consisting of at least one metallic nanostructure and one dielectric compound with externally tunable dielectric properties. From this point of view, SCO complexes are of great interest due to the substantial variation of the real part of their refractive index (*n*) throughout the entire UV, visible and IR frequency ranges. In our research, we proposed a hybrid, SCO–plasmonic device based on gold nanostructures. Employing electron beam lithography (EBL) and lift off strategies, we developed localized surface plasmon (LSP) substrates consisting of a series of arrays of gold nanorods with different aspect ratios (Figure 7a) [34]. After this, the photonic device was finalized with a 60 nm thin film of the SCO complex, Fe(hptrz)_3_(OTs)_2_, spin-coated onto the top.

For a given particle size, aspect ratio and distance between particles, each array of gold nanorods have a characteristic LSP resonance (LSPR) wavelength that will also depend on the refractive index value of the surrounding media (Figure 7b). As a result, the LSPR of these devices can be tuned by varying *n*, due to the spin-state change of the SCO film. Figure 7c displays the LSPR response of the array shown in Figure 7a (covered with a SCO film) as a function of temperature. It was demonstrated that under those experimental conditions, the confined electromagnetic field around the metallic nanostructures can be successfully coupled to the molecular spin state changes brought on by the Fe centers of the SCO film. The LSPR technique was sensitive enough to detect the thermal spin transitions in thin films of up to 60 nm with a conventional optical absorption setup. Furthermore, the spin-state switching behavior was also observed due to plasmonic heating. Such devices that display synergy between plasmon resonance and molecular spin states may be of great interest for implementing detection or self-regulation strategies on-chip for the photothermal effect or, with an appropriate design, even for the development of photonic self-oscillators.

#### Nanoelectronic devices

**Figure 8:**
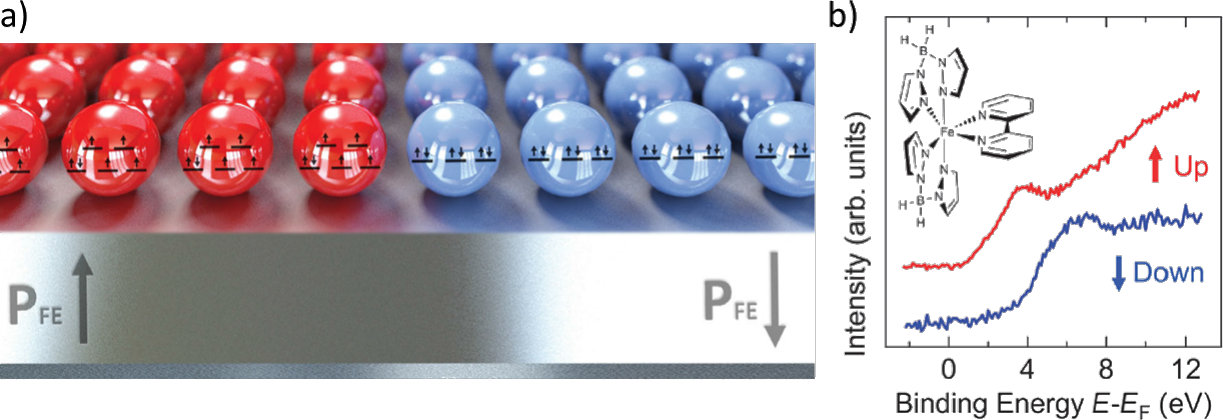
a) Schematic view of the molecular memory proposed by Zhang et al. At low temperatures, the spin state is determined by the polarization of the ferroelectric substrate. If the substrate is poled “up”, the SCO molecules will remain HS; conversely, the SCO molecules will adopt the LS form for a substrate poled “down”. b) Inverse photoemission spectrum at 170 K for a 25 molecule thick layer of [Fe(H_2_B(pz)_2_(bipy)] deposited on PVDF–TrFE poled “up” (red) and “down” (blue). Each spectrum corresponds to the characteristic signature of the complex in the HS and LS form, respectively. Adapted with permission from [35], copyright 2014 The Royal Society of Chemistry.

The act of inducing an SCO transition with an electric field could provide the breakthrough necessary for the development of working molecular memory devices. With this in mind, Zhang et al. designed an experiment where the spin state of a thin film can be controlled by the ferroelectric polarization of the underlying substrate [35]. Through variations observed from inverse photoemeision spectroscopy (IPES) of the film, they inferred the signature of a voltage-controlled, spin-crossover transition that was later validated using magnetometry. For this purpose, 3 nm thick, organic copolymer, ferroelectric polyvinylidene fluoride trifluoroethylene films (PVDF:TrFE, 70:30) were deposited on graphite substrates using the Langmuir–Blodgett technique (Figure 8). Then, an up or down ferroelectric polarization state was preprogrammed into these substrates by scanning a probe (±900 V) before deposition of a SCO layer. The complex [Fe(H_2_B(pz)_2_(bipy)] (pz = pyrazol-1-yl, bipy = 2,2’-bipyridine) was sublimated onto these substrates to form different thin films of 10 to 25 molecular layers in thickness and also onto gold substrates as control samples. It was found that there was a shift in the density of unoccupied states during the spin transition that leads to a significant loss in the density of states just above the Fermi energy level in a HS to LS transition. This situation was observed in SCO films deposited on the gold and the graphite substrates where the ferroelectric PVDF–TrFE film was poled “down”. However, if the interfacial dipoles of the PVDF–TrFE film are poled “up” instead, the characteristic inverse photoemission (IPES) signature of the SCO complex in the HS form persists down to 100 K, well below the thermal spin crossover (Figure 8). These observations in this hybrid device constitute one of the first evidences that electric fields can be effectively employed to address and manipulate spin states in molecular SCO systems on the nanometer scale.

#### Theoretical studies of spin-crossover nano-objects: towards modelling hybrid systems

A reduction in dimensions leads to an inevitable change in the bistability phenomenon for SCO nano-objects [36]. This idea has been experimentally confirmed with the study of spin-crossover nanoparticles [2–5]. The main observations include the shrinking of the thermal hysteresis loop, a downshift in the transition temperature, and an increase of the high spin (HS) residual fraction at low temperature. On the other hand, against all predictions, a surprising cooperative behavior has been observed in very small nanoparticles (2–4 nm) [6–11]. The origin of this effect is not well understood and different explanations can be proposed. For instance, the hysteresis loop in small objects could be the consequence of interactions between particles through the matrix, an interaction between the particle and the matrix [20], or a size dependent variation of the mechanical properties of the particle [37]. Of course all of these phenomena can be coupled, which leads to considerable complexity for the study of size effects in spin-crossover nanoparticles.

In any case, the pervasiveness of the surface-to-volume ratio at the nanometer scale has a major impact on the spin-crossover behavior. Thus, the SCO phenomenon can be controlled by clever engineering of the nanoparticle interface. To this regard, theoretical studies can be very useful to predict the various interface effects. In general, the presence of the surface leads to new energy terms that are added to the internal energy of the system. As a consequence, the internal energy becomes nonextensive, which leads to the modification of the thermodynamic properties of the SCO particles [37–38]. The degree to which the system becomes nonextensive depends on the surface-to-volume ratio. The modulation of the spin-crossover behavior in nanoparticles can be realized by modification of the surface energy terms in the HS and LS states. This energy depends on two different parameters: the energy per surface, γ, and the area of the particle, *A*. The energy per surface term depends on several complicated phenomena which happen at the surface. For instance, coordination defects the surface, the relaxation or reconstruction of the surface, the chemical or physical interaction between the nanoparticle and the environment can all contribute, in a rather complex way, to γ. The theoretical prediction of these phenomena and, even more importantly, their spin-state dependence is currently not possible. On the other hand, the spin-crossover behavior of particles can be altered when their shape is modified [38–39].

**Figure 9:**
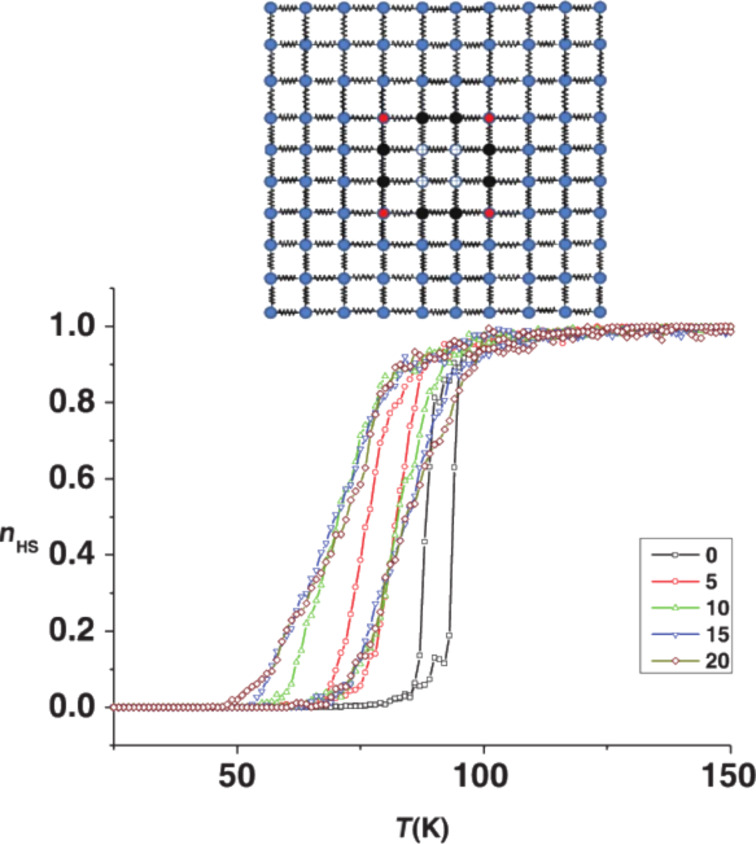
Thermal SCO curves for different thicknesses of an inactive HS shell, calculated with a compressible Ising-like model with harmonic potential. Top panel: core–shell system with an SCO active core and an inactive HS shell. Adapted with permission from [40], copyright 2013 American Physical Society.

**Figure 10:**
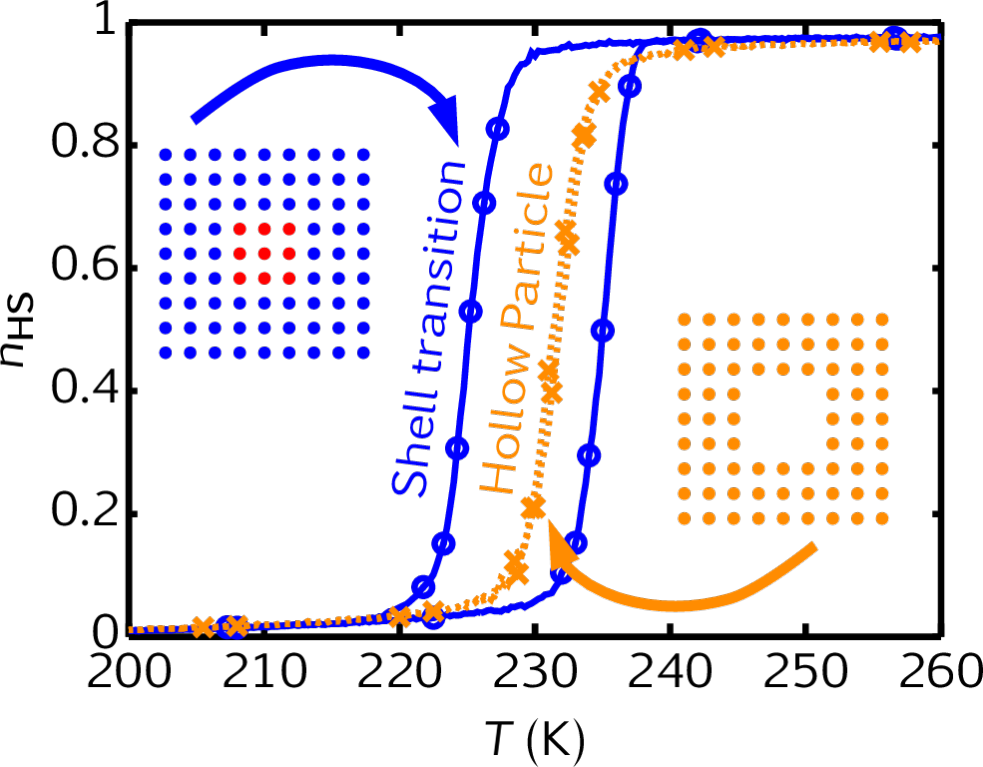
Comparison between the thermal SCO curves of a 9 × 9 hollow particle with a 3 × 3 hole and a 9 × 9 shell surrounding a 3 × 3 active core. Adapted with permission from [41], copyright 2014 Elsevier.

Beyond the modulation of the surface-to-volume ratio, in the case of hybrid core–shell particles, the interaction between the core and the shell can be used to further tune the spin-crossover phenomenon. Oubouchou et al. have shown the impact of an inactive HS shell on an active SCO core [40]. Figure 9 displays thermal SCO curves of a square-shaped SCO core surrounded by 0, 5, 10, 15 and 20 layers of an inactive HS shell. The authors explain the downshift in the transition temperature by a negative elastic pressure applied by the shell, leading to the widening of the thermal hysteresis loop. In another point of view, the downshift in the transition temperature and the cooperative change of the SCO behavior can be understood by additional deformation energies in the internal energy of the core, depending on the core spin state. Félix et al. have shown that the misfit between the lattice parameters of the core and the shell leads to a modification of the spin transition temperature [41]. In the case of an inactive HS shell, the misfit between a LS core and the shell is higher than the misfit between a HS core and the shell. The consequence of this misfit is a downshift in the transition temperature of the core. As shown in Figure 10, the authors have also shown that the synergy between an SCO active shell and an SCO active core leads to a modulation of the SCO behavior, and furthermore, to a new kind of bistability at the nanometer scale.

## Conclusion

The fundamental and technological developments associated with the vast hybrid materials domain are limited only by the imagination of researchers. In the SCO field, thus far, chemists have focused mainly on the elaboration of core–shell or doped, hybrid nanoparticles. While low-level doping is not expected to significantly influence the SCO properties, theoretical models predict important effects in the case of core–shell systems that must be taken into account for the design of such nano-objects. On the other hand, physicists have produced different multilayer structures involving electroluminescent, plasmonic and ferroelectric thin films in interaction with SCO films. The results prove that SCO materials are attractive candidates for integration into photonic and electronic devices. In addition we believe that interesting applications of SCO hybrids can be anticipated in mechanical actuator technology as well. Indeed, the huge spontaneous strain during the spin transition was recently employed by Shepherd et al. through the integration of SCO materials in bimorph cantilevers, which were actuated both thermally and by light irradiation [42]. While these systems used macroscopic materials, in principle, true nanoscale operation is also possible. Exciting strain-induced coupling of SCO with electrical [43] and magnetic properties [44] has also been very recently reported using polymer composite and multilayer heterostructure systems, respectively. Finally, let us note that spintronics may also benefit from SCO nanohybrids as was highlighted by a scanning tunneling microscopy experiment [45].
